# Efficacy of the enhanced recovery after surgery protocol in operating room nursing of patients following single-port video-assisted thoracoscopic lung cancer surgery: A retrospective study

**DOI:** 10.1097/MD.0000000000033427

**Published:** 2023-03-31

**Authors:** Lijun Wei, Yingying Wang

**Affiliations:** a Department of Operation Room, HwaMei Hospital, University of Chinese Academy of Sciences, Ningbo, Zhejiang, China; b Ningbo Institute of Life and Health Industry, Ningbo No. 2 Hospital, University of Chinese Academy of Sciences, Ningbo, Zhejiang, China.

**Keywords:** efficacy, enhanced recovery after surgery protocol, operating room nursing care, single-port video-assisted thoracoscopic lung cancer surgery

## Abstract

This study assessed the efficacy of the enhanced recovery after surgery (ERAS) protocol in operating room nursing care for patients who underwent single-port video-assisted thoracoscopic lung cancer surgery. The study included 82 surgical lung cancer cases. The patients underwent single-port video-assisted thoracoscopic lung cancer surgery between April 1, 2021, and June 31, 2022. Of the 82 patients, 42 received nursing care under the ERAS protocol (experimental group) and 40 had routine nursing care (control group) in the operation room. Based on the 2 different nursing care approaches, the postoperative functional recovery efficacy, quality of life, postoperative complications, and psychological status were compared between the 2 groups. In our analysis, the mean anal venting time, average early out-of-bed time, the average time to liquid resumption, atelectasis, and pulmonary infection rate were significantly lower in the experimental group than in the control group (*P* < .05). The Self-Rating Depression Scale (SDS) and the Self-Rating Anxiety Scale (SAS) scores were also significantly lower in the experimental group than in the control group (*P* < .05). Other indicators were not significantly different between the 2 groups. Our results show that the implementation of an ERAS protocol in operating room nursing care is feasible and should be clinically applied. The ERAS protocol may enhance the recovery of patients who underwent single-port video-assisted thoracoscopic lung cancer surgery.

## 1. Introduction

Lung cancer is a common malignant tumor with a high incidence and mortality worldwide.^[1]^ In recent years, with increasing uptake of physical examinations, an increasing number of pulmonary nodules have been detected through routine computed tomography screening.^[2]^ Therefore, it is reasonable to anticipate that an increasing number of patients with early-stage lung cancer will benefit from timely diagnosis. According to the National Comprehensive Cancer Network guidelines, surgical resection is the primary treatment choice for patients with early-stage lung cancer.^[3]^ To date, perioperative medical research has attracted attention, and several studies have assessed the efficacy of perioperative nursing care in patients undergoing curative surgical resection for the treatment of lung cancer.^[4–6]^ Besides, the indications for single-port thoracoscopic surgery have been extended. At our center, approximately 95% of patients with lung cancer undergo single-port video-assisted thoracoscopic surgery. Some studies have confirmed that single-port video-assisted thoracoscopic surgery performed by experienced surgeons did not increase the risk of postoperative complications, and all patients reported less pain after surgery.^[7,8]^

The concept of enhanced recovery after surgery (ERAS) was introduced for clinical use by a group of surgeons in 1997, and is now widely applied in various surgical procedures.^[9]^ An integrated ERAS protocol consists of adequate preoperative preparations, intraoperative meticulous manipulations, and postoperative close observations. The protocol involves doctors, senior anesthesiologists, and nurses.^[10,11]^ Strengthening ERAS care training is now highly appreciated. Nursing interventions in the operating room as one of the most important parts of the ERAS protocol and have attracted much attention from ERAS study groups.^[11,12]^ According to several studies, the conception of rapid rehabilitation under operating room nursing care of patients undergoing surgery can effectively decrease postoperative stress and potential complications, thus enabling patients to recover as quickly as possible.^[11,13]^ Although conventional operating room nursing care can promote recovery to some extent, it has inherent drawbacks including a focus on the disease itself and the lack of psychological state assessment.^[14]^ The ERAS protocol is receiving increasing attention in operating room nursing care of patients undergoing single-port video-assisted thoracoscopic lung cancer surgery. This study explored the application of ERAS in operating room nursing care for patients who underwent single-port video-assisted thoracoscopic lung cancer in our hospital.

## 2. Materials and methods

### 2.1. General information

A total of 82 patients who underwent single-port video-assisted thoracoscopic lung cancer at our hospital (from April 1, 2021 to June 31, 2022) were enrolled in our study. All patients were diagnosed with lung cancer based on intraoperative histological evaluation, and lobectomy and routine mediastinal lymph node dissection were performed by an experienced thoracic surgeon. The inclusion criteria were as follows. All patients accepted adequate preoperative preparations, and no significant abnormalities were detected before surgery including laboratory examinations and cardiopulmonary function. Age ranged from 16 to 80 years. There were no apparent surgical contraindications. No patient underwent thoracotomy. The exclusion criteria were as follows. Patients who underwent wedge resection, bronchial sleeve resection, or pneumonectomy. Abnormal coagulation test results. The study was reviewed and approved by the Ethics Committee of Hwa Mei Hospital.

### 2.2. Methods

#### 2.2.1. Control group.

All surgeries were performed by an experienced thoracic surgeon. The control group received routine clinical care. Patients were required to fast for 8 hours and abstain from water for 4 hours before surgery. The temperature and humidity of the operating room were maintained within a reasonable range. At the end of surgery and on the postoperative days, the patients received proper and adequate pain relief medications. After discharge, the patients were advised to eat a light diet and return to the outpatient clinic 2 weeks later for review.

#### 2.2.2. Experimental group.

All the surgeries were performed by the same thoracic surgeon. The experimental group was cared for under the ERAS protocol. The workflow involved 3 phases (before surgery, during surgery, and after surgery). Before surgery, nurses familiarized themselves with disease-associated knowledge and the potential complications. Nurses assessed the patient^,^ psychological status and ensured effective communication. Urinary catheters and skin preparations were not indicated for these patients. The patients fasted for 8 hours and abstain from water for 4 hours before surgery. In the operating room, the team of nursing nurses, anesthesiologists, and thoracic surgeons checked the patients’ information, determined body positions, and confirmed the infusion rate. The nurses had the main responsibilities of sterilizing the operative instruments and regulating moderate temperatures in the operating room.^[11]^ Indwelling deep vein catheterization and urinary catheterization were selected according to the surgical method and the patient systemic condition.^[12]^ Deep vein catheterization and urinary catheterization were not indicated when the operation time was short and/or blood loss was limited during surgery. During surgery, more attention is paid to brain protection, body temperature management, and prophylactic antibiotics when the operation time exceeds 3 hours.

Postoperatively, the patients were transferred to the ward. The operating room nurse carefully recorded intraoperative situations, including operation time, blood loss, respiration, circulation, and skin temperature. The operating room nurse also visited the patients on postoperative day 1 and advised the patients to get out of bed as early as possible to promote functional recovery. Meanwhile, patients can drink water after their vitals have been stabilized and gradually transitioned to a light diet. Early resumption of a normal diet can improve nutritional absorption and shorten the hospital stay.^[15]^ All other aspects of care were identical to the control group.

### 2.3. Observational indexes

Data on the intraoperative situation, postoperative functional recovery, postoperative complications, quality of life, and psychological status were retrospectively collected and compared between the 2 groups. The Quality of Recovery-40 questionnaire, which evaluates physical, psychological, and social functioning, was utilized to understand the quality of functional recovery after surgery.^[16,17]^ We also used the Self-Rating Depression Scale (SDS) and the Self-Rating Anxiety Scale (SAS) to predict the patient anxiety or depression symptoms.^[18,19]^ The maximum score were was 100 points for all 3 above-mentioned questionnaires.

### 2.4. Statistical analysis

Data analysis was conducted using the SPSS, version 26.0 (IBM Corp., Armonk, NY). Measurement data were expressed as mean (standard deviation), and the *t* test was used to compare samples or multiple samples for normally distributed data. Enumeration data were expressed as percentages, and the *χ*^2^ test was performed. *P* < .05 was considered statistically significant.

## 3. Results

### 3.1. Clinical indexes

Eighty-two patients were retrospectively analyzed. Based on the different methods of nursing interventions in the operating room, the patients were divided into the ERAS nursing care group (experimental group, n = 42) and the conventional nursing care group (control group, n = 40). All data were normally distributed. No significant differences in age, sex, lesion diameter, or lesion location were found between the 2 groups (*P* > .05) (Table 1).

**Table 1:** Baseline characteristics of all participants.

Variables	ERAS group (n = 42)	Conventional group (n = 40)	*P* value
Age (yr)	50.23 ± 7.03	51.27 ± 8.12	.557
Gender (male/female)	20/22	19/21	.991
Smoking	15	21	.126
Drinking	7	10	.352
Past history [n (%)]			
Diabetes	6 (14.29)	4 (10.00)	.553
Prior history of pulmonary disease	8 (19.05)	11 (27.50)	.363
Lesion diameter (cm)	1.48 ± 0.17	1.65 ± 0.24	.623
Lesion location [n (%)]			.067
Left upper lobe	10 (23.81)	9 (18.33)	
Left lower lobe	7 (16.67)	5 (25.00)	
Right upper lobe	14 (33.30)	13 (33.33)	
Right middle lobe	2 (4.76)	1 (3.33)	
Right lower lobe	9 (21.43)	12 (20.00)	
Pathological type [n (%)]			.737
Squamous carcinoma	7 (16.67)	6 (15.00)	
Adenocarcinoma	33 (78.57)	33 (82.51)	
Others	2 (4.76)	1 (2.50)	
TNM stage [n (%)]			.833
IA	29 (69.05)	28 (70.00)	
IB	6 (14.29)	7 (17.50)	
IIA	2 (4.76)	2 (5.00)	
IIB	1 (2.39)	0 (0.00)	
IIIA	4 (9.52)	2 (5.00)	
CEA (ng/mL)	3.11 ± 0.15	2.85 ± 0.12	.654

CEA = carcinoembryonic antigen, ERAS = enhanced recovery after surgery, TNM = tumor, node, metastasis.

### 3.2. Intraoperative condition

The mean surgical procedure time was longer (*P* = .453) and the number of smooth operations was higher (*P* = .975) in the ERAS group than those in the conventional group, but the difference was not significant. Intraoperative blood loss in the ERAS group was slightly lower than that in the conventional group, but the difference was not significant (*P* = .317) (Table 2).

**Table 2:** Comparison of intraoperative condition.

Variables	ERAS group (n = 42)	Conventional group (n = 40)	*P* value
Mean surgical procedure time (min)	106.45 ± 26.13	103.23 ± 17.56	.453
Intraoperative blood loss (mL)	123.65 ± 36.32	130.54 ± 38.15	.317
Smooth operation [n]	41	39	.975

ERAS = enhanced recovery after surgery.

### 3.3. Postoperative functional recovery situation

The mean anal venting time (*P* = .043), average early out-of-bed time (*P* = .041), and average time to the resumption of liquid (*P* = .0.038) in the ERAS group were markedly lower than those in the conventional group (Table 3). The Quality of Recovery-40 score was significantly higher in the ERAS group than in the conventional group (*P* = .021) (Table 4).

**Table 3:** Comparison of postoperative functional recovery.

Variables	ERAS group (n = 42)	Conventional group (n = 40)	*P* value
Mean anal venting time (h)	19.14 ± 4.23	24.23 ± 5.26	.043
The average early out-of-bed time (h)	17.16 ± 5.32	23.14 ± 6.12	.041
The average time to resumption of liquid (h)	12.15 ± 3.15	17.13 ± 4.08	.038
The QoR-40 score	75.4 ± 3.5	55.3 ± 4.3	.021

ERAS = enhanced recovery after surgery, QoR-40 = Quality of Recovery-40.

**Table 4:** Comparison of postoperative complications of patients between 2 groups.

Variables	ERAS group (n = 42)	Conventional group (n = 40)	*P* value
Cardiac complications	5 (11.9)	6 (15.0)	.681
Atrial fibrillation	3 (7.1)	4 (10.0)	.644
Supraventricular tachycardia	1 (2.4)	1 (2.5)	.975
Sinus bradycardia	1 (2.4)	1 (2.5)	.975
Pulmonary complications	9	19	.013
Atelectasis	1 (2.4)	6 (15)	.041
Pulmonary infection	4 (9.8)	11 (27.5)	.035
Prolonged air leak after operation	4 (9.8)	2 (5)	.432
Incision healing rate	41 (97.6)	40 (100.0)	.326
Total	15 (35.7)	25 (62.5)	.015

ERAS = enhanced recovery after surgery.

### 3.4. Postoperative complications

Perioperative complications included pulmonary, cardiac, and other organic complications.^[20]^ Owing to detailed perioperative care and anesthesia, fatal complications, such as myocardial infarction, cardiac herniation, postoperative thoracic hemorrhage, and pulmonary embolism did not occur. However, the overall complication rate was relatively high. Overall, pulmonary complications, including atelectasis and infection rate, were significantly lower in the ERAS group than in the conventional group (*P* < .05). However, no significant difference was found in cardiac complications or the incision healing rate between the 2 groups.

### 3.5. Postoperative changes in psychological status

Before nursing interventions, there was no significant difference in the SAS and SDS scores between the 2 groups (*P* > .05). After nursing interventions, the SAS and SDS scores of the 2 groups declined. Furthermore, the SDS and SAS scores in the ERAS group were significantly lower than those in the control group (*P* < .05) (Table 5).

**Table 5:** Comparison of psychological status between 2 groups.

Group	SDS score		SAS score	
	Before nursing	After nursing	Before nursing	After nursing
ERAS group	60.05 ± 3.05	24.14 ± 2.42	54.43 ± 4.18	20.12 ± 1.77
*P*	.784	.028	.934	.017

ERAS = enhanced recovery after surgery, SAS = self-anxiety scale, SDS = self-depression scale.

## 4. Discussion

Lung cancer surgery is one of the most common surgeries performed at large hospitals. With the increasing development of surgical techniques and the shortening of surgical learning curves, several complicated lung cancer surgical methods, including broncho-vascular sleeve lobectomy and pneumonectomy, can be completed with a single incision.^[21]^ Currently, hospital stays are generally shorter and hospital costs lower. To maintain these, hospital-based care must be improved. In particular, attentive care is required for a successful surgery. Thus, effective nursing interventions have the potential to improve surgical outcomes and reduce postoperative complications.^[22,23]^

Conventional nursing care predominately focused on curative therapeutic intent and neglected the patient physical and psychological needs, resulting in a less-than-desirable effect. In contrast, patient-centered ERAS not only focuses on the patient interests but also on the needs and wants of the patient.^[24]^ The ERAS protocol is a scientific and advanced nursing care model that can decrease patient stress and surgical injury, thus promoting postoperative recovery and improving patient satisfaction.^[11]^ The ERAS protocol was first introduced and applied at our hospital in 2020. According to recent statistics, the ERAS protocol can effectively reduce total hospitalization costs by 12% and has been validated as an effective method for nursing in our hospital. Currently, the ERAS protocol has gained immense popularity at our hospital.

In our study, the intraoperative conditions between the 2 groups were similar. This may be because one experienced thoracic surgeon completed all the surgeries and was familiar with the surgical procedure. Unexpected intraoperative events during surgery were rare. The mean anal venting time, average early out-of-bed time, and average time to liquid resumption in the ERAS group were reduced. Our results suggest that the ERAS protocol can effectively promote postoperative functional recovery. The reason is that this mode can provide individualized care to every patient and help them complete activities of daily living.

Atelectasis and pulmonary infections are the major postoperative pulmonary complications.^[25]^ Atelectasis is among the most frequent postoperative pulmonary complications and can occur in patients of any age.^[26]^ Atelectasis impairs gas exchange, thus causing hypoxemia and other respiratory disorders, such as acute lung injury and pneumonia. Previous studies have reported that the incidence of atelectasis was significantly lower in the ERAS group.^[27,28]^ Our results were consistent with these studies and atelectasis and other pulmonary complications were significantly lower in the ERAS group.^[27,28]^ Patients were encouraged to get out of bed early, which enabled effective expectoration, thus reducing the incidence rate of postoperative atelectasis and pulmonary infections.^[29]^

This study has some limitations. First, it was based on retrospective data, and thus, causality cannot be inferred. In the future, large-scale multicenter prospective studies are needed to confirm these results and determine whether ERAS directly enhances the recovery of patients after single-port video-assisted thoracoscopic lung cancer surgery. Second, the ERAS protocol was used after a lobectomy in this study. The ERAS protocol should also be validated in other complicated surgical procedures in future studies. Third, the sample size was small. In the future, we intend to conduct a large-scale, multicenter, prospective study to verify our findings.

## 5. Conclusions

In summary, our results showed that the ERAS protocol enhanced the postoperative outcomes. To improve recovery, the ERAS protocol should be considered for patients undergoing single-port video-assisted thoracoscopic lung cancer surgery.

## Author contributions

**Conceptualization:** Yingying Wang.

**Data curation:** Yingying Wang.

**Formal analysis:** Yingying Wang.

**Funding acquisition:** Yingying Wang.

**Investigation:** Yingying Wang.

**Methodology:** Lijun Wei, Yingying Wang.

**Project administration:** Lijun Wei, Yingying Wang.

**Resources:** Lijun Wei, Yingying Wang.

**Software:** Lijun Wei, Yingying Wang.

**Supervision:** Lijun Wei.

**Validation:** Lijun Wei.

**Visualization:** Lijun Wei.

**Writing – original draft:** Lijun Wei.

**Writing – review & editing:** Lijun Wei.
